# Adipocyte death triggers a pro-inflammatory response and induces metabolic activation of resident macrophages

**DOI:** 10.1038/s41419-021-03872-9

**Published:** 2021-06-05

**Authors:** Andreas Lindhorst, Nora Raulien, Peter Wieghofer, Jens Eilers, Fabio M. V. Rossi, Ingo Bechmann, Martin Gericke

**Affiliations:** 1 Institute of Anatomy and Cell Biology, Martin-Luther-University Halle-Wittenberg, Halle (Saale), Germany; 2 Institute of Anatomy, Leipzig University, Leipzig, Germany; 3 Carl-Ludwig Institute of Physiology, Leipzig University, Leipzig, Germany; 4 Biomedical Research Centre, University of British Columbia, Vancouver, Canada

**Keywords:** Diabetes, Obesity

## Abstract

A chronic low-grade inflammation within adipose tissue (AT) seems to be the link between obesity and some of its associated diseases. One hallmark of this AT inflammation is the accumulation of AT macrophages (ATMs) around dead or dying adipocytes, forming so-called crown-like structures (CLS). To investigate the dynamics of CLS and their direct impact on the activation state of ATMs, we established a laser injury model to deplete individual adipocytes in living AT from double reporter mice (GFP-labeled ATMs and tdTomato-labeled adipocytes). Hence, we were able to detect early ATM-adipocyte interactions by live imaging and to determine a precise timeline for CLS formation after adipocyte death. Further, our data indicate metabolic activation and increased lipid metabolism in ATMs upon forming CLS. Most importantly, adipocyte death, even in lean animals under homeostatic conditions, leads to a locally confined inflammation, which is in sharp contrast to other tissues. We identified cell size as cause for the described pro-inflammatory response, as the size of adipocytes is above a critical threshold size for efferocytosis, a process for anti-inflammatory removal of dead cells during tissue homeostasis. Finally, experiments on parabiotic mice verified that adipocyte death leads to a pro-inflammatory response of resident ATMs in vivo, without significant recruitment of blood monocytes. Our data indicate that adipocyte death triggers a unique degradation process and locally induces a metabolically activated ATM phenotype that is globally observed with obesity.

## Introduction

Obesity is linked to numerous diseases, such as atherosclerosis, cancer, cardio-vascular disease, and most prominently diabetes mellitus type 2 (refs. ^1–3^). In clinical practice, body weight or the body mass index are routinely used to classify overweight or obesity^1^. However, metabolically healthy obese individuals indicate that adipose tissue (AT) mass per se is not indicative for development of obesity-associated diseases, but rather an obesity-driven AT dysfunction^2,3^. Hallmarks of AT dysfunction are adipocyte hypertrophy, immune cell infiltration, and increased expression of pro-inflammatory cytokines^2^.

The increase of immune cells in AT of obese individuals during chronic, low-grade inflammation is predominately due to an increase of adipose tissue macrophages (ATMs)^4,5^. ATMs are routinely classified into two groups: ‘classically activated’ (M1) ATMs, that express pro-inflammatory cytokines and decrease insulin sensitivity, and ‘alternatively activated’ (M2) ATMs, that express anti-inflammatory cytokines and participate in tissue remodeling^6^. Resident ATMs in lean mice are mostly M2 polarized, but with progression of obesity, the proportion of M1 ATMs increases^4,7^.

More recent studies, however, found that obesity induced ATM activation differs from classical M1 activation and rather induces a metabolically activated phenotype, characterized by increased lysosomal biogenesis and lipid metabolism^8,9^. A comparable phenotype can be induced by treatment with palmitate, insulin, and glucose (PIG)^10^. Within CLS these activated ATMs digest apoptotic adipocytes via lysosomal exocytosis, resulting in lipid-laden foam cells^8,11^. PIG or obesity-induced metabolic activation of ATMs leads to increased expression of pro-inflammatory cytokines Interleukin (IL) 1β and Tumor necrosis factor (TNF) α^10,12^ and lipid-laden ATMs in CLS, showing pro-inflammatory properties comparable to M1 activation^13^. Consequently, the number of CLS correlates with the progression of AT inflammation^14^.

Initial studies suggested that these pro-inflammatory ATMs are preferentially recruited from blood monocytes^4,15^, but M1 polarization of resident M2 ATMs was also postulated^14,16^. However, the molecular pathways leading to formation and resolution of CLS as well as the origin of pro-inflammatory ATMs remain poorly understood^16,17^.

On the other hand, obesity is characterized by an enhanced turnover of adipocytes^18^. Although the process of differentiation of newly formed adipocytes has been studied intensively in the last years, our knowledge of adipocyte degradation in vivo is rather limited. The degradation process of adipocytes is of special interest, since the number of dying adipocytes increases dramatically in obesity.

In this work, we studied the immune response following adipocyte death. Hence, we introduce the first model for induced cell death of individual adipocytes in living AT by laser irradiation, giving new insights into adipocyte–macrophage interactions following adipocyte death. Our data presented here identify accumulation of multiple ATMs around dying adipocytes as a physiological degradation process leading to a local inflammation, even under homeostatic conditions in lean mice. This is in sharp contrast to fast, anti-inflammatory efferocytosis by single macrophages after cell death of smaller cell types in other tissues. Therefore, analysis of the size-dependent phagocytic capacity of ATMs provides a direct link between adipocyte hypertrophy and AT inflammation.

Our data indicate that adipocyte death is the underlying reason for AT inflammation in obesity. Hence, we here provide first-time evidence for a long-standing hypothesis in AT pathophysiology^14^ and our data, therefore, call for adipocyte-protective strategies in future pharmacotherapy of obesity.

## Results

### Adipocyte size is above the threshold for efficient efferocytosis

**Fig. 1:**
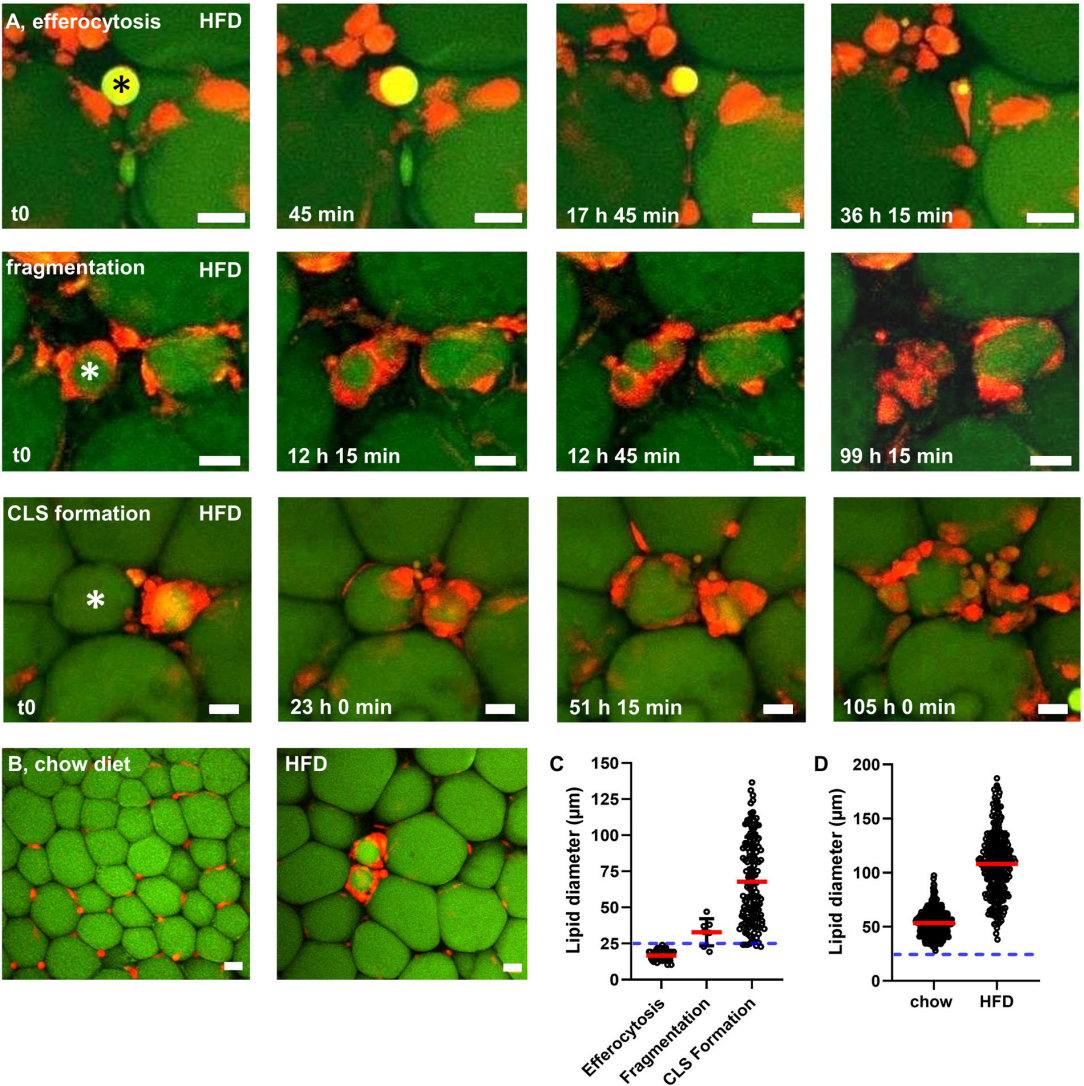
Live-imaging indicates a size threshold for efferocytosis of lipid remnants. Live-imaging of AT explants of HFD-fed MacGreen mice (green: BODIPY-stained lipids, red: ATMs, movies provided as online supplement). **A** Degradation of adipocyte remnants in AT explants occurs in three distinct ways: efferocytosis (upper row), fragmentation (middle row), or CLS formation (lower row). **B** Representative overview images of living AT of chow-fed and HFD-fed mice. **C** Quantification of lipid diameter associated with either efferocytosis, fragmentation, or CLS formation (188 registered events in 40 movies from 6 independent experiments). **D** Quantification of lipid diameter in adipocytes of chow-fed or HFD-fed littermates (*N* = 3). Asterisks mark lipid remnants degraded by ATMs. Blue line indicates the threshold for efferocytosis. Scale bars = 25 µm.

We analyzed ATM activity around lipid droplets (probably resulting from adipocyte rupture) of different sizes in AT explants from HFD-fed mice ex vivo using our established live-imaging approach at single cell resolution. The observed events were recorded and categorized into three groups: classical efferocytosis (uptake of lipid by an individual ATM), fragmentation (separation of one solid lipid remnant into several smaller lipid remnants by ATMs with subsequent uptake) and CLS formation (surrounding of a lipid remnant by several ATMs without evidence for degradation or uptake). Interestingly, the size of lipid remnants affected lipid handling by ATMs. Small lipid droplets were effectively efferocytized and digested by single ATMs (Fig. 1A). In some cases, medium-sized lipid droplets were fragmented into smaller portions and then each small lipid droplet was efferocytized individually by ATMs (Fig. 1A). However, in the vast majority of cases, large lipid droplets could not be efficiently efferocytized or fragmented by ATMs, but instead were surrounded by numerous ATMs, forming characteristic CLS (Fig. 1A). The duration for degradation varied between the described processes. The classical efferocytosis process led to fast digestion of lipid droplets within ~36 h (Fig. 1A). In contrast, fragmentation and digestion took several days (Fig. 1A). The process of CLS formation appeared to take about 24–48 h. However, no uptake or decrease in lipid droplet size was detectable > 100 h after initial ATM-lipid contact in observed cases of CLS formation (Fig. 1A). While we did observe some CLS in AT explants of chow-fed mice, a representative image shows ATMs located between adipocytes, whereas CLS were frequently observed in AT explants of HFD-fed mice (Fig. 1B). To determine a threshold size for the individual lipid-handling processes, we measured the sizes of all lipid droplets cleared or surrounded by ATMs. We found that a diameter of 25 µm is the critical threshold size for classic efferocytosis in living AT (Fig. 1C). Importantly, this threshold for particle uptake was confirmed using BMDMs in vitro and commercially available beads of distinct sizes. BMDMs were able to phagocytose beads up to 20 µm in diameter, whereas phagocytosis of 45 µm beads (similar to small adipocytes) was only rarely observed (Supplementary Fig. 1). In AT ex vivo, some medium-sized lipid droplets between 25 and 50 µm were fragmented and subsequently digested by multiple ATMs. However, most lipid droplets above 25 µm in diameter (presumably dead adipocytes) were surrounded by numerous ATMs to form CLS (Fig. 1C). Next, we measured the lipid droplets of live, unprocessed and unfixed adipocytes in living AT of lean and obese mice. Importantly, we found almost no adipocyte-associated lipid droplet smaller than 25 µm. On average, lipid droplets in adipocytes of chow-fed mice were ~50 µm in diameter. After 20 weeks of HFD, lipid droplets in adipocytes increased in size to an average of ~100 µm, some even reaching close to 200 µm (Fig. 1D). Therefore, our data indicate that almost every case of adipocyte death leaves behind lipid remnants above the critical threshold size for efficient efferocytosis by single ATMs.

### Adipocyte death leads to crown-like structure formation

**Fig. 2:**
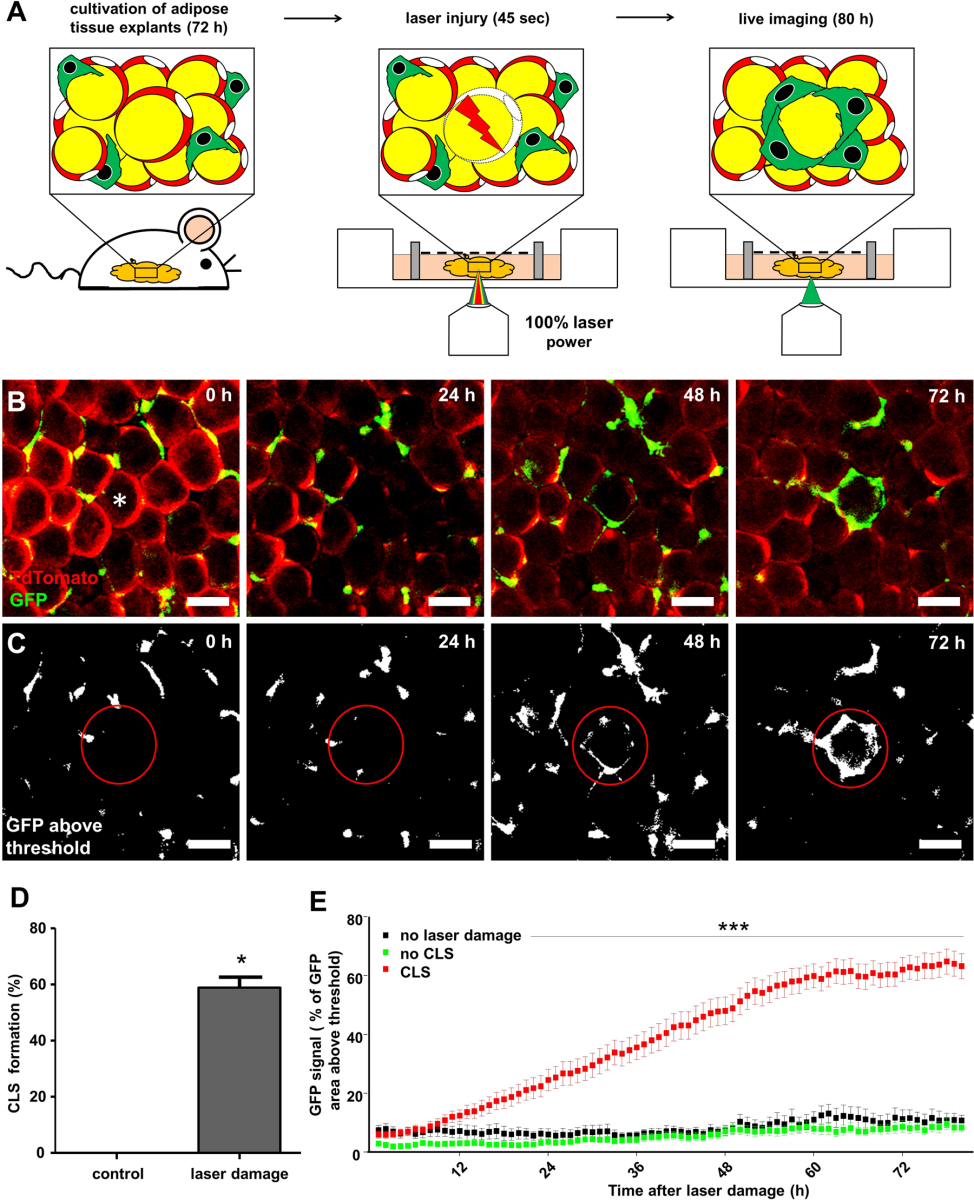
Model of targeted adipocyte death by laser injury in living AT. **A** Scheme of laser injury protocol and **B** formation process of CLS after adipocyte death in AT explants from lean double reporter mice (*Csf1r-*eGFP x *Adipoq*CreERT2:TDTO mice). Movie provided as supplemental online material. **C**, **E** GFP fluorescence quantification in proximity to the targeted adipocyte (**E**, no laser damage *n* = 13; no CLS *n* = 23; CLS formed *n* = 35; *N* = 5). **D** CLS formation after laser injury (*N* = 5). Scale bars = 50 µm. *p < 0.05 and ***p < 0.001. Data presented as mean ± SEM.

Next, we aimed to investigate, if adipocyte death could be a sole cause for CLS formation by targeted depletion of small adipocytes in AT of lean mice. We chose AT from lean mice to exclude interference of an already established inflammation as in AT from obese mice. We established a protocol for precisely killing a single adipocyte by laser injury. After induced adipocyte death, live-imaging was performed for the next 4–5 days (Fig. 2B, C).

In our model, CLS formed around ~60% of target adipocytes, killed by laser injury (Fig. 2D). CLS formation (defined by an ATM-derived increase of GFP fluorescence) started to increase 10 h after laser injury and plateaued after 60 h (Fig. 2E).

**Fig. 3:**
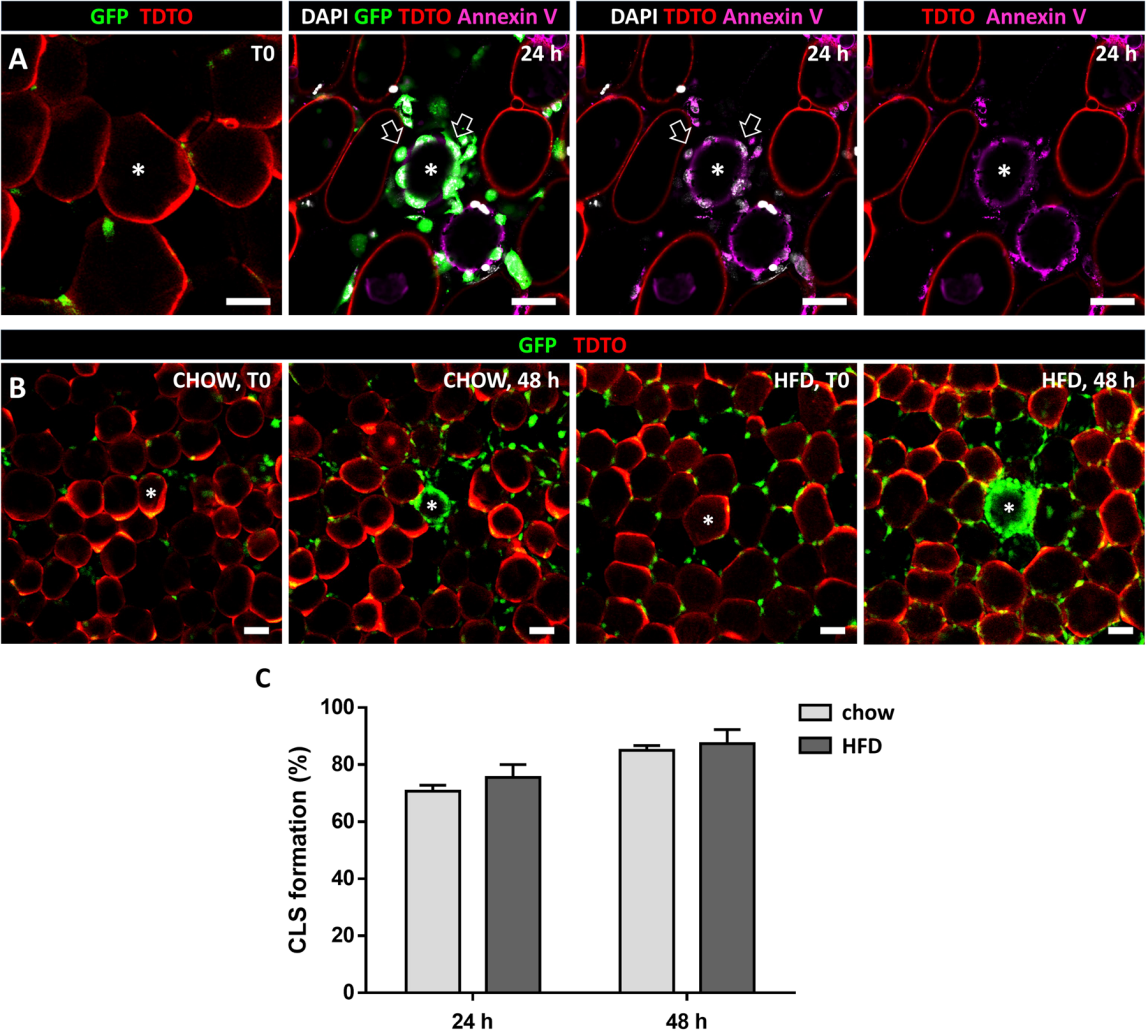
Characterization of adipocyte death and CLS formation post laser injury. **A** Phosphatidylserine externalization detected via Annexin V staining (magenta) 24 h post laser injury. Nucleus of depleted adipocyte shows no DAPI staining, while some surrounding ATMs are DAPI positive (white; highlighted by arrows), validating DAPI staining (*N* = 3). **B** Induced CLS formed 48 h post laser injury in AT explants of chow-fed (chow) and HFD-fed (HFD) mice. **C** Frequency of CLS formation after laser injury (*N* = 3). Asterisks mark depleted adipocytes, scale bars = 50 µm.

Further, we aimed at characterizing the induced adipocyte death and early CLS formation. Phosphatidylserine externalization was detected via Annexin V staining in adipocytes 24 h post laser injury (Fig. 3A). Additionally, nuclei of these adipocytes did not show DAPI staining (Fig. 3A), together indicating an apoptosis-like cell death post laser injury with intact cell membrane at this early stage. Interestingly, CLS formed with the same frequency and within the same timeframe after HFD compared to matched chow-fed controls (Fig. 3B, C).

### Adipocyte death leads to local activation of resident macrophages

**Fig. 4:**
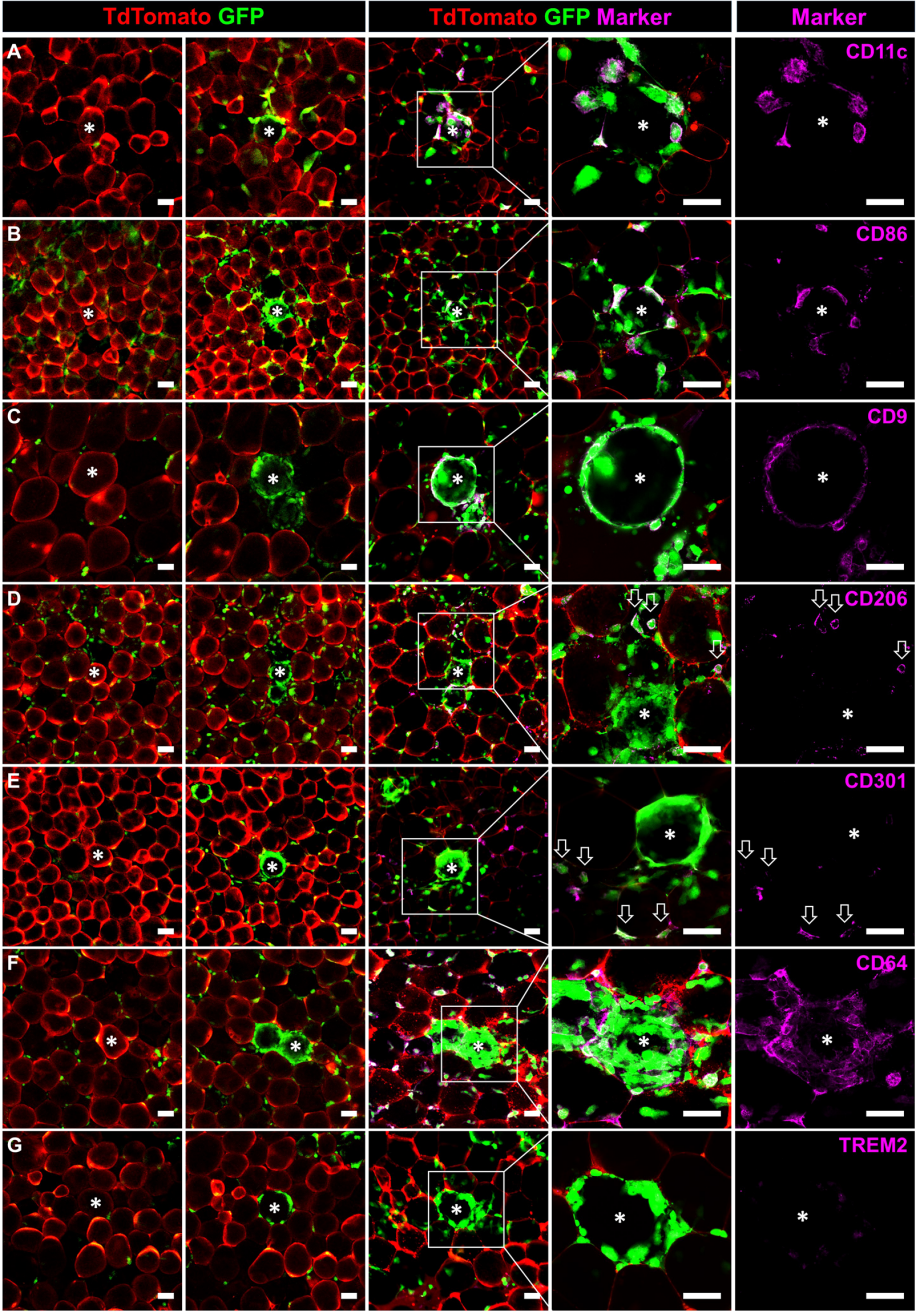
Macrophages in laser-induced CLS exhibit a locally confined M1 polarization. **A**–**C** Expression of M1 markers CD11c, CD86, and CD9 and **D**, **E** M2 markers CD206 and CD301 by ATMs of induced CLS and interstitial macrophages. **F**, **G** Expression of CD64 (all macrophages) or TREM2. Targeted adipocytes are marked by an asterisk. M2 ATMs outside of CLS are highlighted by arrows. Scale bars = 50 µm.

Our live-imaging setup allows for targeted CLS induction and detection in high spatio-temporal resolution. We used this model to further analyze the early immune response of resident ATMs in newly formed CLS by performing post-hoc immunostainings for different M1 and M2 marker proteins. Interestingly, as in obesity, ATMs in CLS were predominantly positive for the commonly used M1 markers CD11c, CD86, and CD9, whereas interstitial ATMs showed almost no expression of these marker proteins (Fig. 4A–C). Staining with the M2 markers CD301 and CD206 mirrored these results as interstitial ATMs were CD301- and CD206-positive, while ATMs in CLS were negative (Fig. 4D, E). Both, interstitial and CLS-forming ATMs showed high expression of CD64 (Fig. 4F), whereas no distinct ATM population with high expression of TREM2 could be detected (Fig. 4G). Of note, the high CD11c expression observed in ATMs forming CLS could not be induced by treatment of ATMs with palmitate or by efferocytosis of small lipid droplets and is, therefore, a unique characteristic for large particle efferocytosis (Supplementary Fig. 2).

### In vivo verification of local activation in induced CLS

**Fig. 5:**
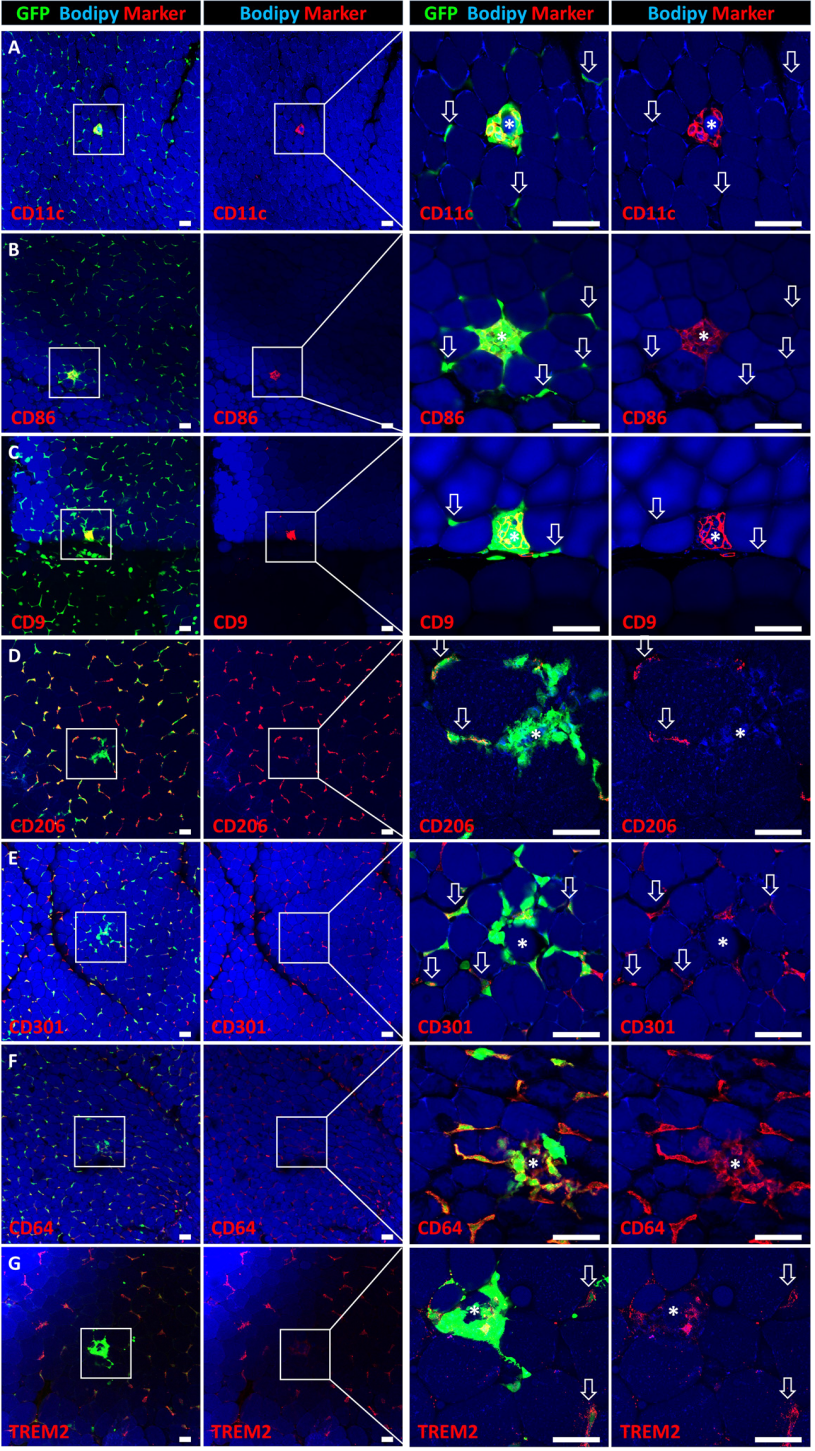
In vivo formed CLS in lean mice show comparable inflammation marker expression to induced CLS. Whole mount antibody staining of CLS formed in vivo in lean mice under homeostatic conditions. **A**–**C** ATMs in in vivo formed CLS express pro-inflammatory markers CD11c, CD86, and CD9, while interstitial ATMs are negative (highlighted by arrows). **D**, **E** Interstitial ATMs express the anti-inflammatory markers CD206 and CD301 (highlighted by arrows), while ATMs in CLS are negative. **F**, **G** Expression of CD64 (all macrophages) or TREM2 (highlighted by arrows). Asterisks mark adipocytes inside CLS. Scale bars = 100 µm.

To exclude that the observed change in M1 – and M2 marker expression is caused by the artificial laser injury and not related to adipocyte death, we also stained AT of lean mice directly after dissection and searched for physiologically occurring CLS due to adipocyte turnover. Interestingly, we obtained similar results compared to laser-induced CLS. CLS-associated ATMs in vivo also showed high expression of putative M1 markers CD11c, CD86, and CD9 (Fig. 5A–C), while interstitial ATMs remain negative (Fig. 5A–C, arrows). Interstitial ATMs expressed the M2 markers CD206 and CD301, in contrast to M2 marker negative CLS-associated ATMs (Fig. 5D, E). Both, interstitial ATMs, and ATMs forming CLS expressed CD64 (Fig. 5F) and TREM2 was expressed in some ATMs forming CLS, but more prominent in interstitial ATMs (Fig. 5G, arrows). All-together, this indicates that the local activation of ATMs in CLS is a physiological response to adipocyte death and no artifact of the used laser-injury model or a consequence of the pro-inflammatory environment in obesity.

### RNA sequencing reveals metabolic activation in CLS

**Fig. 6:**
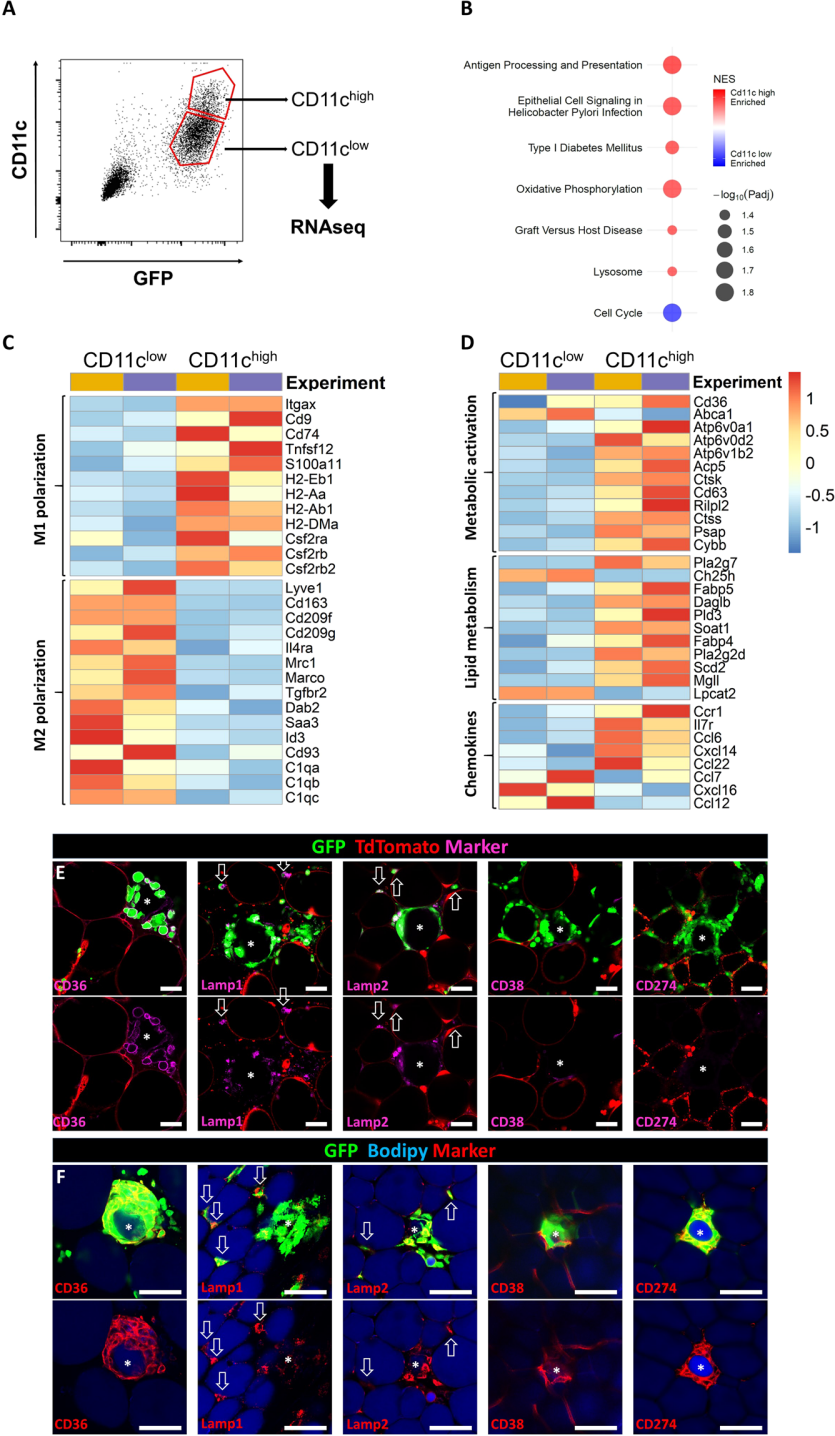
RNA sequencing reveals metabolic activation following adipocyte death. **A** GFP positive cells were sorted in CD11c^high^ and CD11c^low^ groups for RNA sequencing 48 h following laser injury. **B** Pathway analysis of gene expression profiles between the sorted groups. **C**, **D** Heatmaps comparing differentially expressed genes related to either M1 or M2 polarization (**C**) or associated with metabolic activation, lipid metabolism or chemokines and their receptors (**D**). Colors indicate the log_2_ fold change of gene expression in the 2 individual experiments from average across all samples. Expression of genes shown is significantly different between CD11c high and CD11c low group (*p* < 0,001). **E** Expression of markers differentiating between M1 polarization (CD38, CD274) and metabolic activation (CD36) and markers for lysosomal exocytosis in induced CLS. **F** Expression of the respective markers in vivo in lean mice. Asterisks mark adipocytes within CLS, positive interstitial ATMs are highlighted by arrows. Scale bars = 50 µm.

Our results indicated CD11c (*Itgax*) as distinct marker for CLS-associated ATMs early after adipocyte death. Therefore, we sorted ATMs 48 h post laser injury into 2 groups based on CD11c expression and analyzed them using RNA sequencing (Fig. 6A). Pathway analysis revealed increased expression of genes related to antigen presentation and processing, oxidative phosphorylation, and lysosomal biogenesis in the CD11c high group while expression of genes related to cell cycle was decreased (Fig. 6B and Supplementary Table 1). Further, the CD11c high group showed increased expression of several M1 associated genes, while expression of genes associated with a M2 phenotype were significantly reduced (Fig. 6C). CD11c high ATMs also showed significantly increased expression for several genes associated with either metabolic activation or lipid metabolism (Fig. 6D and Supplementary Table 2). Additionally, expression of some chemokines or respective receptors differed between the CD11c high and low ATMs (Fig. 6D), indicating tight regulation of chemotaxis within the tissue. To verify RNA sequencing data on protein level, we proceeded with staining of induced CLS. ATMs in induced CLS expressed CD36, a marker of metabolic activation, while interstitial ATMs were mostly CD36 negative (Fig. 6E). Lamp1 and Lamp2 were also expressed by ATMs in induced CLS, but not exclusively, as interstitial ATMs also showed strong expression as highlighted by arrows (Fig. 6E). The same staining pattern was verified in in vivo formed CLS in lean mice. ATMs residing in CLS express CD36, Lamp1, and Lamp2 (Fig. 6F). Intriguingly, ATMs in induced CLS did not express CD38 and CD274, two selective markers to distinguish metabolic activation from M1 activation, while these markers were strongly expressed in in vivo formed (and probably older) CLS (Fig. 6E, F). This might indicate a time-dependent development of pro-inflammatory characteristics in ATMs forming CLS. Overall, these data reveal that early adipocyte death induces a metabolically activated ATM phenotype that is accompanied by an increased expression of genes associated with M1-polarization.

### Local activation of ATMs is independent of monocyte recruitment

**Fig. 7:**
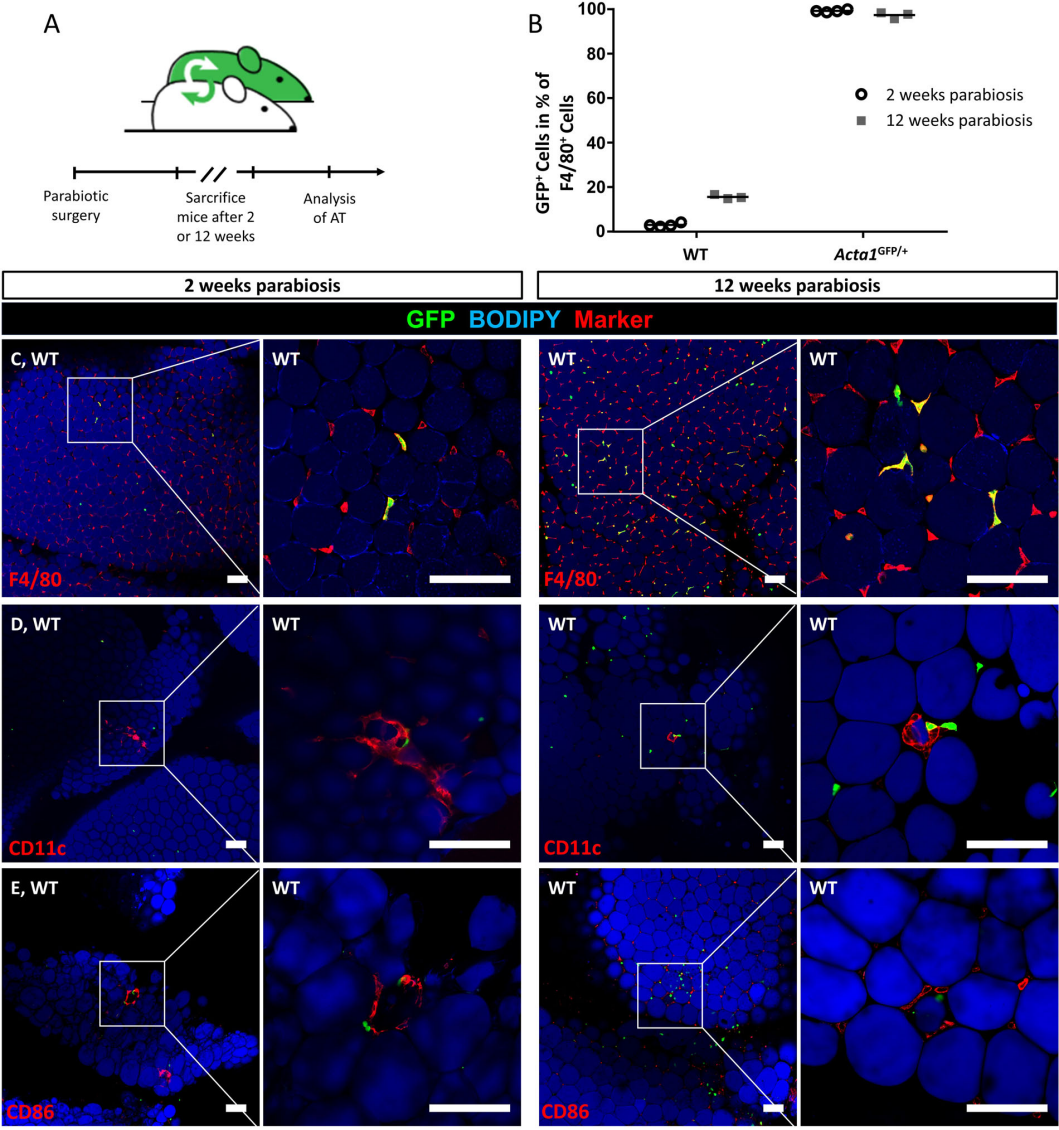
CLS formation does not involve monocyte recruitment in vivo. **A** Timeline of parabiosis experiments. **B** Quantification of GFP^+^ F4/80^+^ cells in AT of *Acta1*^GFP/+^ mice and wild-type parabiotic mice (WT) after 2 weeks (*N* = 4) and 12 weeks (*N* = 3) parabiosis. **C**–**E** Representative images of CD11c and CD86 stained CLS in vivo of WT mice after 2 and 12 weeks parabiosis. Scale bars = 100 µm.

Our previous results indicate that resident ATMs exhibit a metabolic activation with pro-inflammatory properties in response to adipocyte death ex vivo and in vivo. We proceeded to study the involvement of monocyte recruitment to this locally confined AT inflammation around dying adipocytes. Therefore, we performed parabiosis experiments of wild-type and *Acta1*^GFP/+^ reporter mice. The shared blood circulation resulted in ~45% GFP-positive cells in the wildtype parabiotic partner after parabiotic surgery. This allowed for fate mapping of recruited cells from the blood stream into target tissues of the wild-type mice. Importantly, in contrast to the paradigm of monocyte-derived tissue macrophages, very few ATMs were GFP-positive in AT of wild-type mice 2 weeks after parabiosis, increasing to ~20% of GFP-positive ATMs 12 weeks after parabiosis (Fig. 7B, C). Thus, individual ATMs were derived from blood monocytes, but the vast majority of resident ATMs is either extraordinarily long lived or maintained by continuous local proliferation. However, to analyze, whether monocyte recruitment plays a role in physiological CLS formation, we used the M1 markers CD11c and CD86 to stain naturally occurring CLS in lean parabiotic mice (Fig. 7D, E). Although few cells in these in vivo formed CLS were GFP-positive, ~90% of ATMs in CLS were GFP-negative indicating a local origin. Therefore, monocyte recruitment does not seem to have a major impact on CLS formation in vivo.

In conclusion, our data collectively and comprehensively indicate that adipocyte death during adipocyte turnover, even in lean, healthy mice, enhances expression of genes associated with M1-polarization and leads to a metabolic activation of resident ATMs early after adipocyte death.

## Discussion

The link between obesity and associated diseases is AT dysfunction caused by a chronic inflammation^2^. CLS abundance correlates with the progression of AT inflammation leading to accumulation of up to 90% of ATMs in CLS in obese mice^11,25^. It is under debate, whether the pro-inflammatory microenvironment in obesity leads to increased CLS formation or vice versa.

We here used a confocal real-time live-imaging approach to study living AT in order to characterize the dynamics of CLS formation. We further described a new laser injury protocol to induce cell death in individual targeted adipocytes. To the best of our knowledge, this represents the first model to induce single CLS specifically, allowing for a detailed analysis of initial ATM-adipocyte interaction after adipocyte death. Importantly, the timeline of CLS formation after laser injury matches previously proposed data after systemic induction of adipocyte death in vivo^26^. Due to the high spatio-temporal resolution of our approach, the earliest ATM-adipocyte interaction could be observed 10 h following induction of adipocyte death. 24 h post laser injury, membranes of depleted adipocytes were impermeable for DAPI with phosphatidylserine externalization, both features of apoptotic cell death. However both, apoptotic and necrotic adipocyte death can lead to CLS formation, indicating that the cause of adipocyte death does not affect the degradation pathway^11,16,26^. Therefore, our data collectively show that adipocyte death per se induces a locally confined activation of resident ATMs during adipocyte degradation. In contrast, the vast majority of interstitial ATM sustain their anti-inflammatory M2 phenotype in AT explants ex vivo for several days as described before^27^. Thus we directly verify CLS as the center of metabolic activation of ATMs early after adipocyte death accompanied by a shift towards M1-associated gene expression^10,14,16^.

ATMs in induced CLS show increased expression of genes associated with antigen presentation that could indicate involvement of dendritic cells. However, strong expression of CD64 in induced CLS validates these cells as macrophages^28^. TREM2 is discussed as driver in CLS formation and CD11c expression of ATMs in different obesity models^29,30^. We could not detect increased TREM2 expression in CLS-associated ATMs, suggesting that while TREM2 appears to be necessary for CLS formation and adipocyte clearing, it is not upregulated in ATMs forming CLS. In line, others reported that metabolic effects of TREM2 are uncoupled from its expression on ATMs^31^.

Previous studies described monocyte recruitment as key source for pro-inflammatory ATMs in inflamed AT and CLS formation^4,15,32,33^. We here demonstrate that CLS can be induced in AT explants ex vivo (without blood supply), excluding recruitment from blood monocytes as prerequisite for CLS formation and M1 polarization. Most importantly, by analyzing AT from parabiotic mice, we further demonstrate that CLS formation occurs without significant recruitment from blood monocytes in vivo, at least in lean mice. However, ATMs in induced CLS showed changes in expression of chemokines or their receptors, indicating a signaling pathway for chemotaxis of ATMs to the site of adipocyte death within the tissue. Some of the upregulated signaling pathways, such as Ccl22, have been reported to recruit anti-inflammatory macrophages to other tissues, while others, e.g., CXCL14, are implied in recruitment of pro-inflammatory macrophages^34–37^, all-together indicating a tight regulation of intra-tissual recruitment of resident ATMs.

AT inflammation in obesity leads to a distinct metabolic activation of macrophages, characterized by increased lipid metabolism and lysosomal biogenesis^9^ which can be mimicked by treatment with palmitate, insulin, and glucose (PIG)^10^. Importantly, our RNA sequencing results show a similar pattern of upregulated genes by ATMs early after adipocyte death in AT from lean mice. NOX2 (*Cybb*), a key regulator of metabolic activation^12^, is also upregulated early after adipocyte death. This data indicates that adipocyte death suffices to induce a phenotype comparable to obesity-associated AT inflammation or PIG treatment, pointing to adipocyte death as causal link.

In other tissues, homeostatic cell death followed by efferocytosis is an active anti-inflammatory process performed by tissue macrophages^38^. Of note, apoptotic and necrotic adipocyte death both lead to CLS formation^11,16,26^, but in other tissues, necrotic cells are also rapidly cleared by the immune system^39^. Hence, the challenge with efferocytosis of adipocytes appears to be the extraordinary large cell size. We here show, for the first time, that the threshold size for classic, fast efferocytosis by ATMs is ~25 µm. Adipocytes, however, especially after HFD, are up to 5-fold this size, rendering classic phagocytosis impossible. This may lead to longer persistence of cell remnants even after adipocyte apoptosis and a so-called secondary necrosis^39^. Importantly, inefficient efferocytosis is accompanied by increased release of pro-inflammatory cytokines, such as TNFα, whereas release of anti-inflammatory cytokines, like transforming growth factor β and IL-10, is diminished^39–41^. AT inflammation and CLS formation show strong parallels to ineffective efferocytosis, leading to a similar cytokine expression profile^2,5^. Further, both processes show the same cellular cascade, starting with a rapid neutrophil infiltration and resulting in a pro-inflammatory phenotype of tissue macrophages^26^. Therefore, CLS formation is a sign for ineffective efferocytosis of dead adipocytes and we hypothesize that chronic low-grade inflammation in obesity is a result of this process.

PIG treatment leads to NOX2-mediated increased expression of pro-inflammatory cytokines IL1β, IL6, and TNFα^12^. While our data does not show increased expression of these cytokines in induced CLS, we believe that at later time points after CLS induction, this will be the case, as NOX2 is already upregulated in induced CLS and CD11c expressing ATMs are strongly associated to pro-inflammatory cytokine release^42^. In line, early CLS after laser injury did not express CD38 or CD274 (selective markers for M1 activation)^10^, whereas CLS in vivo (which are probably older) strongly express both proteins. This suggests a progressive pro-inflammatory phenotype of ATMs within CLS and stresses the importance of fast and effective clearing of dead adipocytes.

While ATMs in induced CLS show many similarities to LPS stimulated M1 macrophages, their metabolism differs profoundly. Our data show increased expression of genes associated with oxidative phosphorylation, that is usually attributed to M2 macrophages, while classically activated macrophages increase glycolysis and decrease oxidative phosphorylation^43^. This further questions the classic M1/M2 dogma and underlines that CLS-associated ATMs have a unique phenotype.

In summary, our results indicate adipocyte death as the major reason for CLS formation and a subsequent metabolic activation of ATMs that shows pro-inflammatory characteristics analogous to inefficient efferocytosis, as origin for chronic inflammation in obesity. Obesity induced hypertrophy, increased metabolic rate, hypoxia and oxidative stress in visceral AT lead to increased turnover of adipocytes and, therefore, more CLS formation as well as longer abundance of CLS until complete adipocyte clearing. Thus, expanding the locally confined inflammation around individual dead adipocytes to chronically inflamed, dysfunctional AT. Importantly, since our data indicate that adipocyte death is the underlying reason for AT inflammation in obesity, adipocyte-protective strategies in future pharmaco-therapy should be considered.

## Methods

### Mice

Animal experiments followed the ‘Principles of laboratory animal care’ (NIH publication no. 85e23, revised 1985) as well as specific national laws approved by the local authorities. Mice were housed in pathogen-free facilities in groups of three to five mice at 22 ± 2 °C on a 12-h light/dark cycle. Mice were fed either a standard chow diet or a high fat diet (HFD, both Sniff GmbH, Soest, Germany) and had free access to water. For visualizing macrophages in vivo, we used *Csf1r*-eGFP reporter mice (MacGreen mice)^19^. For live-imaging of adipocytes–macrophage interactions, MacGreen mice were crossed with *Adipoq*CreER^T2^*:Rosa26-tdTomato*^*flox/flox*^ mice^20^. Of note, these mice exhibit previously reported sufficient tamoxifen-independent tdTomato (TDTO) expression, especially in homozygous mice^21^. Therefore, no tamoxifen induction was applied.

### Adipose tissue explant culture

For CLS induction, we used an established tissue culture model^19^. After sacrifice, the rostral part of the epididymal white adipose tissue (EWAT) was dissected and cut into small pieces of <1 mm³ (AT explants) under sterile conditions at 37 °C in PBS. AT explants were then transferred to six-well plates filled with 1 ml RPMI cell culture medium supplemented with 10% fetal bovine serum, a 1% insulin-transferrin-selenium mixture (1.0 mg/ml bovine insulin, 0.55 mg/ml human transferrin (iron-free), and 0.5 µg/ml sodium selenite (Sigma-Alderich, Munich, Germany) and antibiotics (100 U/ml penicillin and streptomycin). AT was immobilized at the bottom of wells by sterile cell culture inserts (Merck Millipore, Darmstadt, Germany). Five AT explants per well were cultured at 37 °C with 5% CO_2_ and 21% O_2_ for 3 days before starting live-imaging experiments. During cultivation, the medium was regularly exchanged carefully without removing cell culture inserts.

### Live-imaging of naturally occurring CLS in AT explants of HFD-fed mice

Live-imaging was performed on AT explants of HFD-fed MacGreen mice stained with 1 µg/ml BODIPY 558/568 C12 (Life Technologies GmbH, Darmstadt, Germany) as described previously^19^. To quantify morphologically distinct ways of lipid degradation, 188 such events were registered in 40 multi-day movies (mean observation time > 6 d) from 6 independent experiments. In these movies, ATM–lipid interactions were classified post-hoc into three classes: efferocytosis (full engulfment and subsequent intracellular degradation), fragmentation (separation of a big lipid droplet into two or more small lipid droplets with subsequent efferocytosis), or CLS formation (attachment of multiple ATMs to a central lipid droplet without signs of degradation). After classification, the lipid droplet diameter was measured in the last image before the respective event occurred. For direct comparison, living AT explants of chow-fed and HFD-fed MacGreen littermates were stained and imaged in the exact same manner as described above to determine the mean lipid droplet diameter within living adipocytes without artefacts from fixation, tissue dehydration, or embedding.

### Laser-injury model to induce adipocyte death in AT explants of chow-fed mice

Laser-injury of individual adipocytes was performed on AT explants of chow-fed mice without pre-established AT inflammation. After the 72 h of cultivation, the six-well plate was transferred into a preheated (37 °C, 5% CO_2_) incubation chamber on a confocal FV300 microscope (Olympus Deutschland GmbH, Hamburg, Germany). 200 µm stacks with 10 µm slices were collected once per hour for ~80 h. Adipocyte death was induced by a laser injury. We used a 20x objective lens for laser injury with 10x zoom on the supposed nucleus (identified by adipocyte sickle ring-like shape) irradiating with the available laser lines at 100% intensity for 45 s using the “point scan” mode (used lasers and respective power: 405 nm: 4 mW; 491 nm: 0.42 mW; 553 nm: 15 µW; 633 nm: 0.62 mW). One single adipocyte was irradiated per explant. For quantification, stacks were flattened using the “max intensity” mode and a region of interest (ROI) was determined around the irradiated adipocyte (Fig. 2). Then, the GFP positive area above a determined threshold within the ROI was measured. Threshold was set to exclude unspecific background noise, ensuring specificity of the GFP signal. Formation quantification analyses were performed using Fiji software 2.0 (ref. ^21^).

### Whole mount staining

Antibody staining was performed on AT whole mounts and AT explants after live-imaging. Of note, AT explants were immobilized on the cell culture inserts by adding a drop of histoacryl (B. Braun Melsungen AG, Melsungen, Germany) on top of the respective inserts, keeping AT explants in position without interfering with the antibody staining. AT explants fixed to inserts were then washed with PBS and subsequently fixated in zinc formalin (Polysciences, Hirschberg, Germany) for 15 min. For whole mount staining of living mice, EWAT was dissected immediately after sacrifice, washed in PBS, fixated for 20 min in zinc formalin and cut into small pieces (<1 mm³). After fixation, the tissue was washed with PBS, blocked with staining buffer (3% bovine serum albumin (BSA) in PBS) for 1 h, and stained with pre-labeled antibodies in staining buffer (1:100 for antibodies from BioLegend, San Diego, USA: CD9 [Cat# 124810], CD36 [Cat# 102610], CD38 [Cat# 102716], CD11c [Cat# 117312], CD64 [Cat# 139332], CD86 [Cat# 105020], CD274 [Cat# 124312], F4/80 [Cat# 123122] and from R&DSystems, Minneapolis, USA: TREM2 [Cat# FAB17291A]; 1:200 for antibodies from AbD serotec, BioRad, Feldkirchen, Germany: CD301 [Cat# MCA2392A647] and CD206 [Cat# MCA2235A647T] at 4 °C overnight. For whole mounts of mice with no TDTO^+^ adipocytes, we used 1 µg/ml BODIPY 558/568 C12 and Hoechst (1:10,000 in PBS, Life Technologies) to stain neutral lipids of adipocytes and nuclei, respectively. Samples were then washed 3 times with PBS, transferred into cavities of microscope slides, and mounted using fluorescent mounting medium (Dako; Hamburg, Germany). AT explants fixed to inserts, were cut off together with the adherent filter membrane after the last washing step. Finally, the AT explant was mounted with the tissue-side facing the cover slip, allowing imaging from the exact same region as during live-imaging. Also, in some cases AT explants fixed to inserts were transferred to glass-bottom six-well plates with 1 ml PBS and imaged immediately without fixation. For apoptosis detection, iFluor 647 labeled Annexin V assay was used (Abcam, Cambridge, UK). Explants were immobilized on inserts 24 h after laser injury using histoacryl as described before. After buffer rinse, explants were stained with 1 µg/ml DAPI (Thermo Fischer Scientific) in PBS for 30 min, washed twice with assay buffer, transferred to glass-bottom six-well plates with 1:100 Annexin V in assay buffer and imaged immediately. Images were acquired using an inverted FV1000 confocal microscope (Olympus).

### Culture of bone marrow-derived macrophages (BMDMs) and phagocytosis assay

Bone marrow was flushed with PBS from femurs and tibiae of adult female MacGreen mice. BMDMs were differentiated in RPMI1640 (Thermo Fisher Scientific) supplemented with 10 mM glucose (Sigma-Aldrich), 1 mM GlutaMax, 10% FBS, 1% Penicillin/Streptomycin (all from Thermo Fisher), and 20 ng/ml M-CSF (PeproTech, Hamburg, Germany). At day 3 and 5 fresh M-CSF and culture media was added and cells were harvested at day 7 with ice-cold 1 mM EDTA/PBS solution. BMDMs were seeded at a density of 3 × 10^5^/ml in a 12-well plate in RPMI1640 (10% FBS and 1% Pen/Strep). After two hours, cells were incubated for 48 h with IgG-opsonized and BODIPY-stained beads (Polysciences, Hirschberg, Germany) with 10 µm (600.000/well), 20 µm (150.000/well), or 45 µm (30.000/well) diameter. Experiments were performed in duplicates. Representative images were acquired at a confocal FV1000 microscope (Olympus) to ensure full engulfment of the phagocytosed beads. Quantification of cell suspensions was performed using a MACSQuant 16 analyzer (Miltenyi Biotech, Bergisch Gladbach, Germany). Cells were harvested with Cell dissociation solution (5 min at 37 °C, Sigma), washed with staining buffer (3% BSA in PBS), and analyzed directly following addition of DAPI (0.2 µg/ml) for dead cell exclusion. Quantification of phagocytosis was performed at DAPI-negative, GFP-positive single cells using the increasing SSC as a readout for phagocytosis. Uptake of BODIPY-stained beads was further verified by increasing BODIPY signal in phagocytosing BMDMs (Supplementary Fig. 1C).

### Small lipid efferocytosis

AT was cultivated in explants as above in RPMI medium additionally containing 1 µg/ml BODIPY 558/568 C12. After a 24 h cultivation period, AT explants were washed in PBS and fixated for 15 min in zinc formalin, washed in PBS, blocked with staining buffer for 1 h at room temperature, and stained with a pre-labeled CD11c antibody (BioLegend, San Diego, USA) 1:100 in staining buffer at 4 °C overnight. AT explants were then washed three times with PBS and mounted in cavities of microscopic slices using fluorescent mounting medium. AT explants were cultivated from chow-fed mice as well as from mice having received a HFD for 12 or 24 weeks, respectively.

### Collagenase digestion

We used collagenase to digest AT and extract the stromal vascular fraction. Adipose tissue was chopped up with scissors and digested by 1 mg/ml (~315 U/ml) collagenase type 2 (Worthington, Lakewood, USA) in digestion buffer (13 mM HEPES; 0,8 mM ZnCL_2_; 3% BSA in HBSS) shaking at 1400 rpm at 37 °C for 20 min. Digestion was stopped by adding 10% FBS and tubes were put on ice. The suspension was filtered using 70 µm mesh filter and centrifuged twice (400 *g*, 5 min, 4 °C, no break) after adding staining buffer.

### Palmitate stimulation

AT of male C57BL6 mice was cultivated using the tissue explant model as described above. After 72 h, different concentrations of palmitate were added to the cell culture medium. After 24 h cultivation with palmitate, the AT explants were digested using collagenase as described above. After the last centrifugation step, the cell pellet was resuspended in staining buffer and Fc-receptors blocked by anti-CD16/32 treatment (1:100, eBioscience, Frankfurt, Germany) for 10 min on ice. After centrifuging (300 *g*, 5 min, 4 °C, no break), the pellet was resuspended in staining buffer and stained with 1:100 dilution of F4/80-AF488 and CD11c-AF647 (BioLegend, San Diego, USA) for 20 min on ice. Cells were then centrifuged (300 *g*, 5 min 4 °C), resuspended in staining buffer and kept on ice until analysis. DAPI was added to exclude dead cells. CD11c-positive cells and mean fluorescence intensity of F4/80 positive, living, single cells were analyzed using a MACSQuant 16 analyzer.

### Cell sorting and RNA sequencing

48 h post laser injury, AT explants were digested using collagenase as described above. Cells were resuspended in staining buffer and the Fc-receptors blocked by anti CD16/32 (1:100, eBioscience, Frankfurt, Germany) treatment for 10 min on ice. After centrifugation (300 *g*, 5 min, 4 °C, no break) cells were stained with pre-labeled CD11c antibody (BioLegend, San Diego, USA) on ice for 15 min, centrifugated (300 *g*, 5 min, 4 °C, no break), resuspended in cultivation medium with Hoechst (1:10.000), and kept on ice until sorting. Living, GFP-positive cells were sorted into a CD11c high and a CD11c low group (Fig. 6A, ~10,000 cells per sample) using a BD FACSAria SORP (Becton Dickinson, Franklin Lakes, USA) and frozen at −80 °C in TRIzol (Thermo Fischer Scientific) until sequencing. RNA sequencing and analysis was performed by Single Cell Discoveries (Utrecht, The Netherlands). RNA extraction and library preparation followed the CEL-seq2 protocol^22^ with a sequencing depth of 10 million reads/sample. Results were then analyzed using the DESeq2 package on Rstudio^23^. Gene set enrichment analysis was performed with datasets from The Molecular Signatures Database. Shown is the KEGG pathway analysis of the C2 gene set.

### Parabiosis experiments

We thank Fabio M. V. Rossi for kindly providing AT from parabiotic mice. Pairs of WT and *Acta1*^GFP/+^ mice were surgically connected for two and twelve weeks as previously described^24^. Blood samples were evaluated by flow cytometry at the day of dissection to verify a successful blood sharing of 45.7 ± 2.6% (mean ± SEM) of GFP^+^/CD45^+^ cells in the wild-type parabiotic partner.

### Statistical analysis

Data are presented as means ± SEM or as dot plots of at least three independent experiments or mice were evaluated. Data were checked for normality by using GraphPad Prism (GraphPad Software Inc., La Jolla, USA) and subsequently analyzed by either a two-tailed Student’s *t* test or Mann–Whitney *U*-test. *P* values < 0.05 were considered statistically significant. Sample size was estimated based on previous experiments. For qualitative analyses at least three independent experiments were performed. Compared groups showed similar variances. Analyses were performed without additional blinding of the respective investigator. Allocation to the groups were done according to the genotyping without randomization.

## Supplementary information

Supplemental Figure 1

Supplemental Figure 2

Supplemental Information

Supplemental Table 1

Supplemental Table 2

Supplemental Movie 1

Supplemental Movie 2

Supplemental Movie 3

Supplemental Movie 4
