# Supplementary figures and images for: Adipocyte death triggers a pro-inflammatory response and induces metabolic activation of resident macrophages
Source: Cell Death Dis. 2021 Jun 5;12(6):579. doi: 10.1038/s41419-021-03872-9 (PMC8179930; doi:10.1038/s41419-021-03872-9)

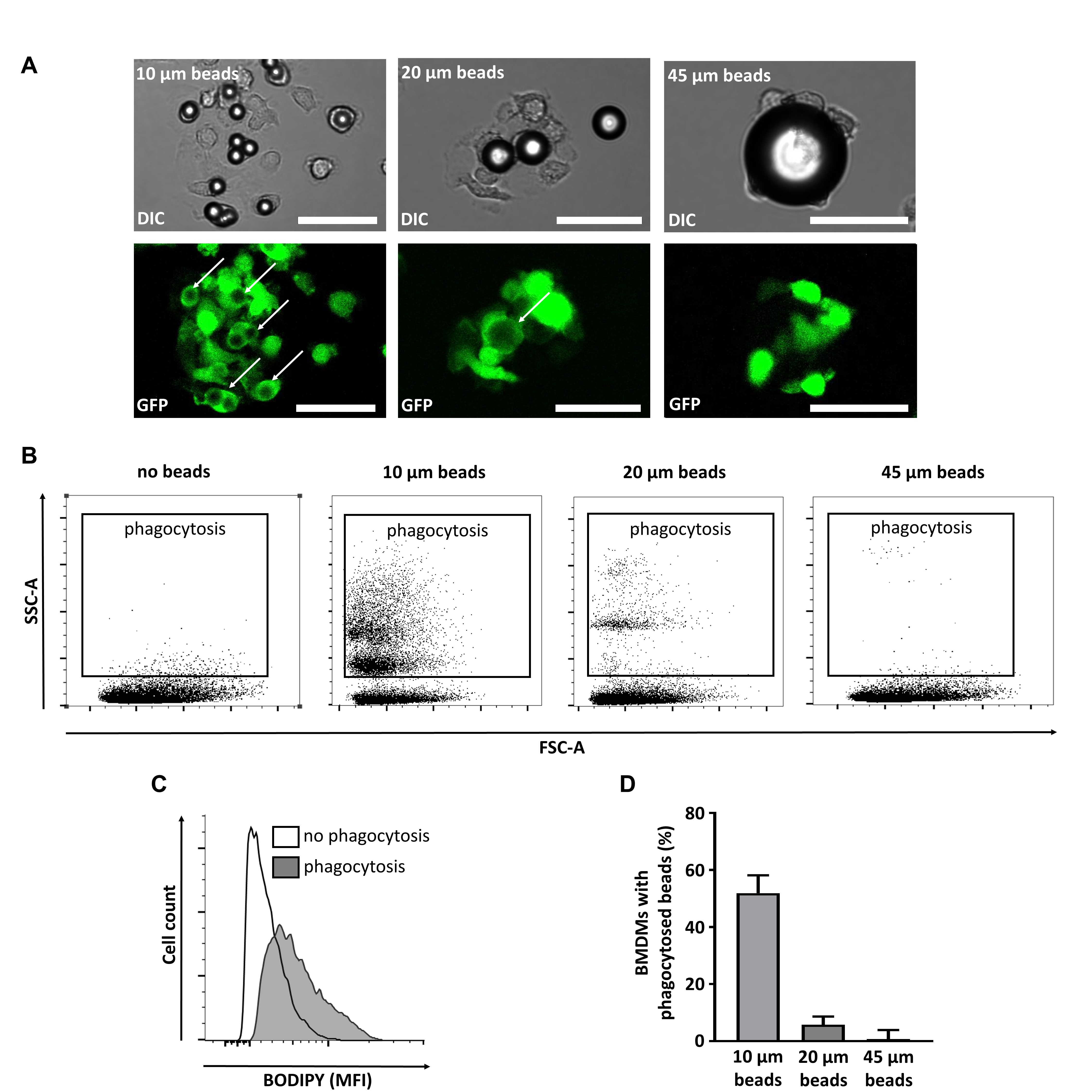

Supplement: Supplementary file 1 — Supplemental Figure 1 [file 41419_2021_3872_MOESM1_ESM.jpg]

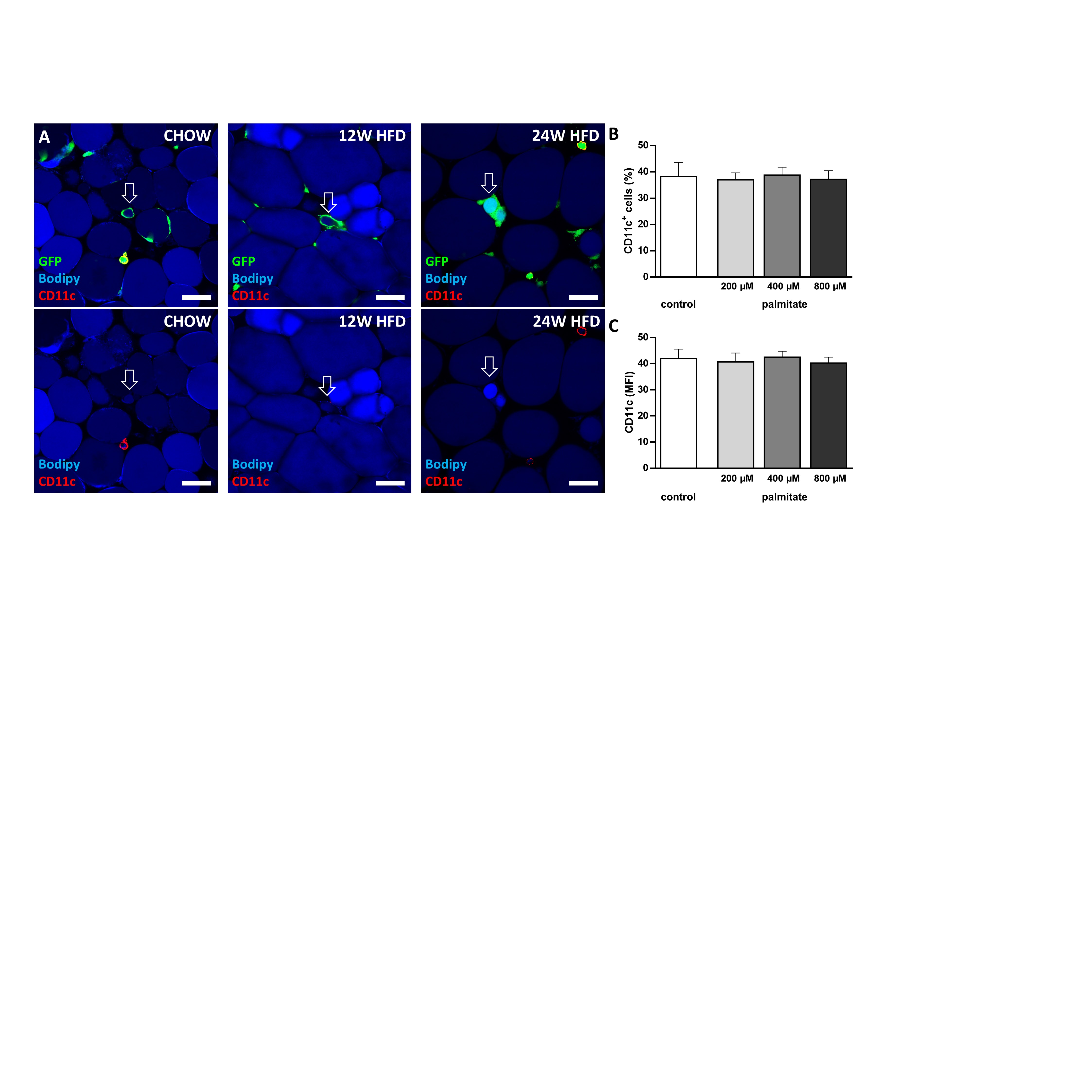

Supplement: Supplementary file 2 — Supplemental Figure 2 [file 41419_2021_3872_MOESM2_ESM.jpg]
