# Supplementary material for: Adipocyte death triggers a pro-inflammatory response and induces metabolic activation of resident macrophages
Source: Cell Death Dis. 2021 Jun 5;12(6):579. doi: 10.1038/s41419-021-03872-9 (PMC8179930; doi:10.1038/s41419-021-03872-9)
Supplement: Supplementary file 3 — Supplemental Information [file 41419_2021_3872_MOESM3_ESM.docx]

**Supplemental information**

Supplemental Figure 1: Size-dependent phagocytosis by bone marrow-derived macrophages

(A) Phagocytosis of 10 and 20 µm beads by BMDMs of MacGreen mice. Of note, 45 µm beads could not be phagocytized. Lack of GFP fluorescence in cytoplasm of ATMs indicates phagocytosis (arrows). (B) Representative flow cytometry plots of BMDMs after incubation with beads of the indicated sizes. (C) Histogram shows BODIPY-signal in BMDMs indicating uptake of 10 µm BODIPY-stained beads. (D) Quantification of phagocytosis of different indicated bead sizes using FACS analysis (B-D). Scale bars = 50 µm.

Supplemental Figure 2: Efferocytosis of small lipid droplets or fatty acid stimulation does not induce CD11c expression in macrophages

(A) In AT explants, ATM sustain CD11c-negative phenotype following efferocytosis of small lipid droplets (<25 µm; highlighted by arrows). (B, C) Palmitate challenge did not increase the number of CD11c positive ATMs or mean fluorescence intensity (MFI) of CD11c. Scale bars = 50 µm.

Supplemental Table 1: KEGG pathway analysis of sorted and sequenced CD11c high vs. CD11c low ATMs following adipocyte death induction

Supplemental Table 2: List of significantly regulated genes of CD11c high vs. CD11c low ATMs following adipocyte death induction by RNAseq

Supplemental Movies

Supplemental movies 1-3 show the 3 processes of lipid degradation depicted in figure 1.

Supplemental Movie 4 shows exemplary live imaging footage of laser injury and induced crown-like structure formation.
